# 96319 Al-Anon Intensive Referral (AIR): A qualitative formative evaluation for implementation

**DOI:** 10.1017/cts.2021.543

**Published:** 2021-03-30

**Authors:** Jure Baloh, Geoffrey M. Curran, Christine Timko, Kathleen M. Grant, Michael A. Cucciare

**Affiliations:** 1 University of Arkansas for Medical Sciences; 2 University of Arkansas for Medical Sciences, Central Arkansas Veterans Healthcare System; 3 VA Palo Alto Health Care System, Stanford University School of Medicine; 4 VA Nebraska-Western Iowa Healthcare System (Omaha), University of Nebraska Medical Center

## Abstract

ABSTRACT IMPACT: This formative evaluation can inform selection and development of implementation strategies for implementing this and other similar interventions in future implementation studies or practice. OBJECTIVES/GOALS: Al-Anon mutual-help groups help concerned others (COs; e.g., families, friends) of persons with an alcohol use disorder better cope with their own problems. Despite widespread availability of Al-Anon meetings, participation is limited. We developed and evaluated an intervention to facilitate CO engagement in Al-Anon. METHODS/STUDY POPULATION: Al-Anon Intensive Referral (AIR) was developed to facilitate COs’ engagement in Al-Anon through four coaching sessions and is being tested in a NIAAA-funded randomized controlled trial (RCT). Consistent with a hybrid type 1 effectiveness-implementation design, we also conducted a formative evaluation to learn about facilitators, barriers and recommendations for AIR implementation in substance use disorder (SUD) treatment programs. We interviewed key informants (director and two staff) at eight sites in the AIR RCT and two ‘naive’ sites unfamiliar with AIR. Sites included community and Veterans Administration (VA) treatment programs in Arkansas, California, and Nebraska. Semi-structured interviews were based on the Consolidated Framework for Implementation Research, and were thematically analyzed. RESULTS/ANTICIPATED RESULTS: Facilitators included AIR’s face validity, adaptability, and alignment with staff values and skills, requiring only minimal training. Several community sites thought AIR would fit with their current practices (e.g. family groups), and some sites reported having sufficient staff available for delivering AIR. Barriers included limited staff time (some sites), and VA sites having limited resources for providing services to COs. Furthermore, many clients have no COs, or COs who are unwilling or unable to engage. Recommendations included fitting AIR within existing workflows and focusing on COs with highest readiness. Participants also thought AIR could be adapted as an online or smartphone app, which may expand its reach to younger and more tech-savvy populations while decreasing staff burden. DISCUSSION/SIGNIFICANCE OF FINDINGS: AIR has strong potential for implementation, but sites vary on implementation capacity and readiness. Most sites could implement it partially (e.g., case-by-case basis), and sites with sufficient capacity (e.g., family groups, staff time) could implement it more fully. An app-based AIR could help mitigate some barriers.

